# Severity of Cognitive Impairment Moderates the Effect of Depression on Motivation

**DOI:** 10.1093/geroni/igaa057.946

**Published:** 2020-12-16

**Authors:** Claire Growney, Xianghe Zhu, Julia Sorensen, Emily Smith, Shevaun Neupert

**Affiliations:** North Carolina State University, Raleigh, North Carolina, United States

## Abstract

Motivation to engage in cognitive activities is a critical feature of cognitive health, especially for older adults. A growing body of literature demonstrates that personal resources such as mental health contribute to older adults’ motivation. However, little is known about factors that contribute to motivation for cognitive engagement in older adults already experiencing significant decline, a population for whom engagement may be uniquely consequential. In the present study, older adults (age 61-85) with cognitive impairment (Short Blessed scores of 7+) and/or dementia diagnoses completed a series of assessments of cognitive ability, well-being, and motivation to engage in cognitively demanding activities as a part of a larger project. Linear regression analyses revealed that cognitive ability moderated the effect of depression on motivation to engage in cognitively demanding activities. For those with relatively mild levels of cognitive impairment, depression was associated with lower levels motivation, whereas depression was unrelated to motivation for those with more severe cognitive impairment. Notably, results were unchanged when controlling for physical health, suggesting that depression has unique effects on motivation for these individuals. Results contribute to a growing body of literature demonstrating that mental health is particularly consequential for individuals in earlier stages of cognitive decline. We suggest that interventions targeting mental health in individuals beginning to experience cognitive decline may be effective in increasing motivation to engage in activities that are beneficial for cognitive health and that such interventions may be most effective when implemented at the earliest stages of dementia.

